# Red blood cell distribution width as long-term prognostic markers in patients with coronary artery disease undergoing percutaneous coronary intervention

**DOI:** 10.1186/s12944-019-1082-8

**Published:** 2019-06-12

**Authors:** Ting-Ting Wu, Ying-Ying Zheng, Xian-Geng Hou, Yi Yang, Xiang Ma, Yi-Tong Ma, Xiang Xie

**Affiliations:** 1 Department of Cardiology, First Affiliated Hospital of Xinjiang Medical University, No. 137, Liyushan Road, Urumqi, 830011 People’s Republic of China; 2 Department of Cardiology, First Affiliated Hospital of Zhengzhou University, Zhengzhou, 450052 People’s Republic of China

**Keywords:** Red blood cell distribution width, Mortality, Coronary artery disease, Percutaneous coronary intervention

## Abstract

**Background:**

The aim of this study was to assess the prognostic value of red blood cell distribution width (RDW) in patients with coronary artery disease undergoing percutaneous coronary intervention (PCI).

**Methods:**

A retrospective cohort study (CORFCHD-PCI, [Identifier: ChiCTR-INR-16010153]) of 6050 patients who were hospitalized with a diagnosis of coronary artery disease (CAD) and treated with PCI from January 2008 to December 2016 were enrolled in the study. The primary outcome was long-term mortality after PCI. Clinical follow-up data of participating patients were obtained during an outpatient examination 35.9 ± 22.6 months after PCI. Demographic and clinical data and admission laboratory parameters were recorded, and patients were categorized into two groups according to RDW level (high group ≥13.1%; low group < 13.1%).

**Results:**

Multivariate Cox regression analysis revealed RDW as an independent prognosis factor for cardiac death. The incidence of cardiac death increased 1.33 times in patients in the high RDW group (HR, 1.331; 95% CI, 1.009–1.755, *P* = 0.043). Kaplan–Meier survival analysis suggested that patients with high RDW tended to have an increased accumulated risk of cardiac death. However, we did not found significant differences in the incidence of long-term mortality (adjusted HR = 1.203[0.941–1.537], *P* = 0.140), MACCE (adjusted HR = 1.128[0.979–1.301], *P* = 0.096), MACE (adjusted HR = 1.155[0.994–1.341], *P* = 0.059), stroke, bleeding events or readmission between the two groups.

**Conclusion:**

The baseline level of RDW is an independent predictor for cardiac death in post-PCI CAD patients.

## Introduction

Coronary artery disease (CAD) is a complicated multifactor disease. A large number of recent clinical and basic research found that patients with CAD exhibit the traditional risk factors and some newly identified risk factors, such as new lipid parameters, atherogenic index of plasma, nonhigh-density lipoprotein cholesterol and the apolipoprotein B to apolipoprotein A1 ratio [1–3]. Peripheral blood parameters were also identified, such as platelet distribution width, mean platelet volume, hemoglobin and serum total bilirubin [4–6], and several inflammatory parameters were demonstrated, such as C-reactive protein, interleukin, fibrinogen, Cystatin C, and high homocysteine levels [7–9].

Biomarkers have become integral to the care of patients with CAD, and sometimes these markers are the key to establishing a diagnosis, determining risk, and guiding therapy. As one of the characteristic parameters of blood, red blood cell distribution width (RDW) was only used for the diagnosis and differential diagnosis of anemia before 2007. Felker et al. [10] found that RDW could be used as an independent predictor of the prognosis of patients with heart failure. A growing number of research gradually found that RDW was closely related to the prognosis of cardiovascular disease. The association between increased RDW and heart failure [11–13], atrial fibrillation [14–16], and pulmonary hypertension [17, 18] emerged in some studies. The relationship between RDW and coronary heart disease was initially studied recently [19–21]. However, there are relatively few studies investigating the prognosis values of RDW in patients with PCI. Therefore, our study examined the RDW levels of patients with CAD undergoing PCI and discussed the effect of RDW level on long-term clinical outcome.

## Methods

### Design and study population

The Clinical Outcomes and Risk Factors of Patients with Coronary Heart Disease after PCI (CORFCHD-PCI) study is a large, single-center retrospective cohort study based on case records and a follow-up registry performed in the First Affiliated.

Hospital of Xinjiang Medical University. The details of the design are registered at http://www.chictr.org.cn (Identifier: ChiCTR-INR-16010153). The CORFCHD-PCI study was designed to evaluate the clinical outcomes and risk factors.

of CAD patients after PCI. We collected demographic data, clinical characteristics, risk factors, blood samples, biochemical data, electrocardiographs (ECG), echocardiography, coronary angiography, PCI procedure, and long-term outcomes for CAD patients who underwent PCI in the First Affiliated Hospital of Xinjiang Medical University from January 2008 to December 2016. The study protocol was approved by the ethics committee of the First Affiliated Hospital of Xinjiang Medical University. Because of the retrospective design of the study, the need to obtain informed consent from eligible patients was waived by the ethics committee. Follow-up data were obtained via review of the medical records and/or telephone interview with the patient or family members.

### Definitions

Hypertension was defined as a systolic blood pressure of > 140 mmHg and/or a diastolic blood pressure of > 90 mmHg in at least 2 measurements or the use of any antihypertensive drug. Diabetes mellitus was defined as fasting plasma glucose levels of 126 mg/dl on multiple measurements or current use of anti-diabetic medications. Hypercholesterolemia was considered total serum cholesterol of > 200 mg/dl or the use of lipid-lowering medication. Smoking and drinking status was defined as current tobacco and alcohol use.

### Clinical and demographic characteristics

Peripheral venous blood samples of the patients were obtained on admission in the inpatient ward. Data on clinical and demographic characteristics, including age, sex, history of hypertension and diabetes mellitus, and smoking and drinking status, were collected from medical records. Echocardiography and laboratory data, including complete blood cell count, lipid parameters and left ventricular ejection fraction (LVEF) were noted. During the follow-up period, the use of β-blockers, angiotensin-converting enzyme inhibitors (ACEIs), angiotensin II receptor blockers (ARBs), statins, aspirins, clopidgrel and calcium channel blockers (CCBs) were recorded.

### Endpoints

The primary endpoint of the study was long-term mortality, including all-cause mortality (ACM) and cardiac mortality. The key secondary endpoints were stroke, bleeding events, readmission and major adverse cardiac events (MACE), which were defined as the combination of cardiac death, recurrent myocardial infarction, and target vessel reconstruction as described previously [20]. Briefly, deaths were considered a cardiac condition unless a definite cause of noncardiac was identified. Recurrent myocardial infarction was defined as a new Q wave, and an increased concentration of creatine kinase MB to greater than 5 times the upper limit of the normal range within 48 h after procedure or new Q waves or an increase in creatine kinase MB concentration to greater than the upper limit of the normal range plus ischemic symptoms or signs, if occurring more than 48 h after the procedure, as described previously [20]. Stroke is defined as a sudden onset of vertigo, numbness, aphasia, or dysarthria caused by cerebrovascular disease, including hemorrhage, embolism, thrombosis, or aneurysm rupture, and persisting for > 24 h [20]. Bleeding events were determined according to the Bleeding Academic Research Consortium (BARC) standard [21]. Target vessel revascularization (TVR) was defined as any repetitive revascularization in a treated vessel where there was at least a 50% diameter stenosis in the presence of ischemic signs or symptoms or at least 70% stenosis in the absence of ischemic signs or symptoms [20]. An adjudication committee that was blinded to the patient group determined all incidents. An event adjudication committee that was blinded to patient group adjudicated all events.

### Statistical analysis

SPSS 22.0 Statistical Package Program for Windows (SPSS Inc., Chicago, Illinois) was used for all statistical analysis. The study population was divided into two groups based on RDW level. The Kolmogorov-Smirnov test was used to assess normality of distribution. Continuous variables with a normal distribution are reported as the mean ± standard deviation, and categorical variables are specified as numbers and percentages. To compare parametric continuous variables, Student’s t tests were used, and to compare nonparametric continuous variables, Mann-Whitney U tests were used. Chi-squared (χ^2^) tests were used to compare categorical variables. Multivariate Cox regression analysis was used for determinations of independent parameters for prognosis. Sequential models were developed to examine the incremental prognostic value of the parameters. Incremental factors added to the model at each step were considered significant when the difference in the log-likelihood associated with each model corresponded to *P* < 0.05. Long-term survival was analyzed using the Kaplan-Meier method. The *P* values were 2-sided, and *P* < 0.05 was considered significant.

## Results

**Fig. 1:**
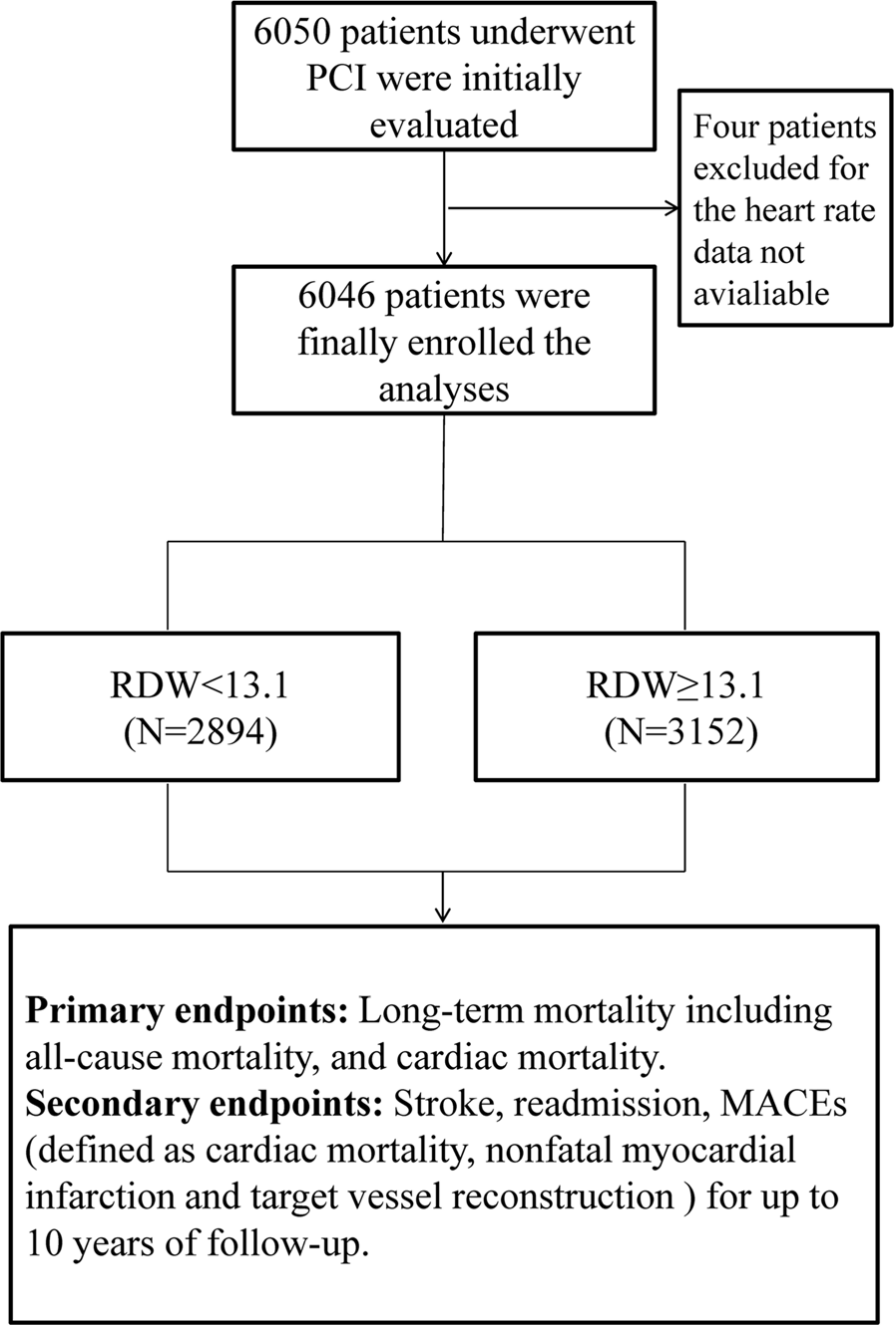
The follow chart of participants inclusion

The follow-up charts of participants are shown in Fig. 1**.** The mean follow-up was 35.9 ± 22.6 months. RDW values ranged from 9.00 to 21.60% (median = 13.1%; interquartile range 12.60–13.60%). We divided RDW into two groups according to the median. Baseline characteristics of the two groups are shown in Table 1. There were 2894 patients in the low RDW group and 3152 patients in the high RDW group. Participants with higher RDW values were more likely to be older with a higher hypertension incidence, higher heart ratio (HR), systolic blood pressure (SBP), blood urea nitrogen (BUN), and creatinine (Cr) and lower use of statins and aspirin (*P* < 0.05). High proportions of males, high rate of drinking, smoking, and diabetes incidence and higher fasting blood glucose (FBG) were found in the low RDW group (*P* < 0.05).

**Table 1:** Baseline characteristics

Variables	Low RDW (*n* = 2894)	High RDW (*n* = 3152)	*P* value
Age (years)	57.8 ± 10.7	60.9 ± 10.7	< 0.0001
Male [n (%)]	2215 (76.5%)	2280 (72.3%)	< 0.0001
Smoking [n (%)]	1236 (42.7%)	1183 (37.5%)	< 0.0001
Drinking [n (%)]	925 (32.0%)	841 (26.7%)	< 0.0001
Hypertension [n (%)]	1169 (40.4%)	1386 (44%)	0.005
Diabetes [n (%)]	738 (25.5%)	713 (22.6%)	0.009
BMI (kg/m^2^)	25.91 ± 7.36	26.01 ± 7.06	0.744
SBP (mmHg)	126 ± 18	127 ± 19	0.006
HR (bpm)	75 ± 11	76 ± 11	0.041
WBC(×10^9^/l)	7.39 ± 2.42	7.34 ± 2.41	0.417
RDW(%)	12.54 ± 0.39	13.84 ± 0.90	< 0.0001
PLT(×10^9^/l)	216 ± 58	214 ± 61	0.283
BUN (mmol/l)	5.38 ± 1.55	5.65 ± 1.78	< 0.0001
Cr (mmol/l)	74.69 ± 18.39	77.02 ± 22.09	< 0.0001
UA (mmol/l)	321 ± 88	325 ± 92	0.068
FBG (mmol/l)	6.81 ± 3.79	6.39 ± 3.03	< 0.0001
TG (mmol/l)	1.92 ± 1.25	1.88 ± 1.28	0.234
TC (mmol/l)	3.94 ± 1.09	3.97 ± 1.12	0.241
HDL-C (mmol/l)	1.01 ± 0.44	1.02 ± 0.51	0.264
LDL-C (mmol/l)	2.43 ± 0.89	2.48 ± 0.93	0.090
ApoA-I(g/l)	1.16 ± 0.30	1.16 ± 0.32	0.511
ApoB(g/l)	0.84 ± 0.35	0.86 ± 0.43	0.123
Gensini Score	31 ± 36	33 ± 42	0.182
LVEF(%)	61 ± 7	61 ± 7	0.464
Prior Medication			
Beta-blocker [n (%)]	1175 (40.8%)	1251 (39.8%)	0.436
ACEI or ARB [n (%)]	658 (22.9%)	708 (22.6%)	0.780
Statin [n (%)]	1637 (57.1%)	1619 (51.8%)	< 0.0001
Aspirin [n (%)]	2014 (70.1%)	2034 (64.9%)	< 0.0001
Clopidgrel [n (%)]	898 (31.3%)	938 (29.9%)	0.245
CCB [n (%)]	347 (12.1%)	343 (10.9%)	0.171

*ACEI* angiotensin-converting enzyme inhibitor, *ARB* angiotensin receptor antagonist, *CCB* calcium channel blocker, *LVEF* left ventricular ejection fraction, *SBP* systolic blood pressure, *HR* heart ratio, *WBC* white blood cell, *RDW* red cell distribution width, *BUN* blood urea nitrogen, *Cr* creatinine, *UA* Uric Acid, *FBG* fasting blood glucose, *TG* triglyceride, *TC* total cholesterol, *HDL-C* high-density lipoprotein cholesterol, *LDL-C* low-density lipoprotein cholesterol, *ApoA-I* apolipoprotein AI, *ApoB* apolipoprotein B

The incidence of total primary end point was 3.9% in the low RDW group and 6.2% in the high RDW group (*P* < 0.001). The incidences of readmission, main adverse cardiovascular and cerebrovascular events (MACCE) MACE, cardiac death and recurrent myocardial infarction were also significantly different between the two groups (*P* < 0.05), and significant increases were observed in the higher group. Although there was a significant difference in the use rate of aspirin between the two groups, we did not find differences in the incidence of bleeding events or stroke between the two groups (Table 2).

**Table 2:** Long term cardiac events

Clinical outcomes	Low RDW (*n* = 2894)	High RDW (*n* = 3152)	*P* value
Primary end point			
Long-term mortality, n (%)	113 (3.9%)	196 (6.2%)	< 0.0001
Secondary endpoints			
Stroke, n (%)	35 (1.2%)	47 (1.5%)	0.344
Readmission, n (%)	360 (12.4%)	459 (14.6%)	0.016
MACEs, n (%)	317 (11.0%)	468 (14.8%)	< 0.0001
MACCEs, n (%)	351 (12.1%)	511 (16.2%)	< 0.0001
Cardiac death, n (%)	88 (3.0%)	163 (5.2%)	< 0.0001
Stroke, n (%)	35 (1.2%)	47 (1.5%)	0.344
Bleeding events, n (%)	76 (2.6%)	99 (3.1%)	0.233
Re-stent implantation	118 (4.1%)	143 (4.5%)	0.380
Recurrent MI, n (%)	79 (2.7%)	115 (3.6%)	0.043

*MACCEs* main adverse cardiovascular and cerebrovascular events, *MACEs* major adverse cardiac events, *Recurrent MI* recurrent myocardial infarction

Univariate models for each of the predictor variables were created, and variables that were significant (*P* < 0.05) in univariate Cox models were entered into multivariate Cox regression analysis. In multivariate Cox regression analysis, after adjusting for the traditional clinical prognostic factors, including age, sex, diabetes, hypertension, smoking, drinking, SBP, HR, BUN, Cr, FBG, use of statins, aspirin, and clopidgrel, which were the significant factors in univariate Cox models, we found that RDW also predicted poor clinical outcomes. RDW was an independent predictor for cardiac death in the high RDW group. The incidence of cardiac death increased 1.33 times (HR, 1.331; 95% CI, 1.009–1.755, *P* = 0.043) (Table 3). However, we did not find significant differences in long-term mortality (adjusted HR = 1.203[0.941–1.537], *P* = 0.140), MACCE (adjusted HR = 1.128[0.979–1.301], *P* = 0.096, Tables 4 and 5), MACE (adjusted HR = 1.155[0.994–1.341], *P* = 0.059), recurrent myocardial infarction (adjusted HR = 1.208[0.897–1.628, *P* = 0.214), stroke (adjusted HR = 1.009[0.641–1590], *P* = 0.968), bleeding events (adjusted HR = 1.068 [0.780–1.463], *P* = 0.681) or readmission (adjusted HR = 1.012[0.877–1.168], *P* = 0.867) among two groups (Tables not shown).

**Table 3:** Cox regression analysis results for cardiac death

Variables	B	SE	X^2^	*P* value	HR (95%CI)
Age	0.016	0.007	5.581	0.018	1.016 (1.003–1.029)
Sex	−0.050	0.166	0.091	0.763	0.951 (0.686–1.318)
Smoking	−0.101	0.173	0.345	0.557	0.904 (0.644–1.267)
Drinking	0.097	0.182	0.283	0.595	1.102 (0.771–1.574)
Diabetes	0.343	0.163	4.447	0.035	1.409 (1.024–1.938)
Hypertension	−0.040	0.142	0.078	0.780	0.961 (0.728–1.269)
Statin	−0.911	0.234	15.125	< 0.001	0.402 (0.254–0.636)
Aspirin	−2.022	0.215	88.559	< 0.001	0.132 (0.087–0.202)
Clopidgrel	−1.282	0.292	19.284	< 0.001	0.278 (0.157–0.492)
SBP	−0.006	0.004	2.560	0.110	0.994 (0.987–1.001)
HR	0.026	0.005	24.149	< 0.001	1.026 (1.016–1.037)
BUN	0.099	0.038	6.756	0.009	1.104 (1.025–1.189)
Cr	0.004	0.003	1.677	0.195	1.004 (0.998–1.010)
FBG	−0.038	0.025	2.284	0.131	0.963 (0.916–1.011)
RDW high vs. low	0.286	0.141	4.100	0.043	1.331 (1.009–1.755)

**Table 4:** Cox regression analysis results for long-term mortality

Variables	B	SE	X^2^	*P* value	HR (95%CI)
Age	0.024	0.006	15.765	< 0.001	1.024 (1.012–1.036)
Sex	−0.051	0.150	0.188	0.731	0.950 (0.708–1.274)
Smoking	0.011	0.153	0.006	0.940	1.012 (0.749–1.366)
Drinking	0.046	0.163	0.081	0.776	1.047 (0.761–1.441)
Diabetes	0.172	0.148	1.394	0.246	1.188 (0.888–1.589)
Hypertension	0.041	0.126	0.105	0.746	1.042 (0.813–1.335)
Statin	−0.140	0.222	22.039	< 0.001	0.353 (0.229–0.546)
Aspirin	−2.072	0.199	108.629	< 0.001	0.126 (0.085–0.186)
Clopidgrel	−1.308	0.271	23.377	< 0.001	0.270 (0.159–0.459)
SBP	−0.005	0.003	2.745	0.098	0.995 (0.989–1.001)
HR	0.022	0.005	21.653	< 0.001	1.023 (1.013–1.032)
BUN	0.081	0.034	5.467	0.019	1.084 (1.013–1.160)
Cr	0.003	0.003	0.963	0.326	1.003 (0.997–1.008)
FBG	−0.013	0.021	0.376	0.540	0.987 (0.947–1.029)
RDW high vs. low	0.185	0.125	2.179	0.140	1.203 (0.941–1.537)

**Table 5:** Cox regression analysis results for MACCEs

Variables	B	SE	X^2^	*P* value	HR (95%CI)
Age	0.000	0.004	0.000	0.999	1.000 (0.993–1.007)
Sex	−0.129	0.094	1. 901	0.168	0.879 (0.731–1.056)
Smoking	−0.129	0.090	2.065	0.151	0.879 (0.737–1.048)
Drinking	−0.081	0.094	0.735	0.391	0.922 (0.766–1.110)
Diabetes	0.193	0.086	5.048	0.025	1.212 (1.025–1.434)
Hypertension	0.231	0.075	9.564	0.002	1.260 (1.088–1.459)
Statin	−0.036	0.081	0.203	0.652	0.964 (0.823–1.130)
Aspirin	−0.502	0.079	40.160	< 0.001	0.605 (0.518–0.707)
Clopidgrel	0.360	0.077	22.005	< 0.001	1.433 (1.233–1.665)
SBP	−0.001	0.002	0.440	0.570	0.999 (0.995–1.003)
HR	0.014	0.003	21.255	< 0.001	1.014 (1.008–1.020)
BUN	0.050	0.022	5.406	0.020	1.052 (1.008–1.097)
Cr	−0.001	0.002	0.092	0.762	0.999 (0.996–1.003)
FBG	−0.005	0.012	0.191	0.662	0.995 (0.972–1.018)
RDW high vs. low	0.121	0.073	2.768	0.096	1.128 (0.979–1.301)

**Fig. 2:**
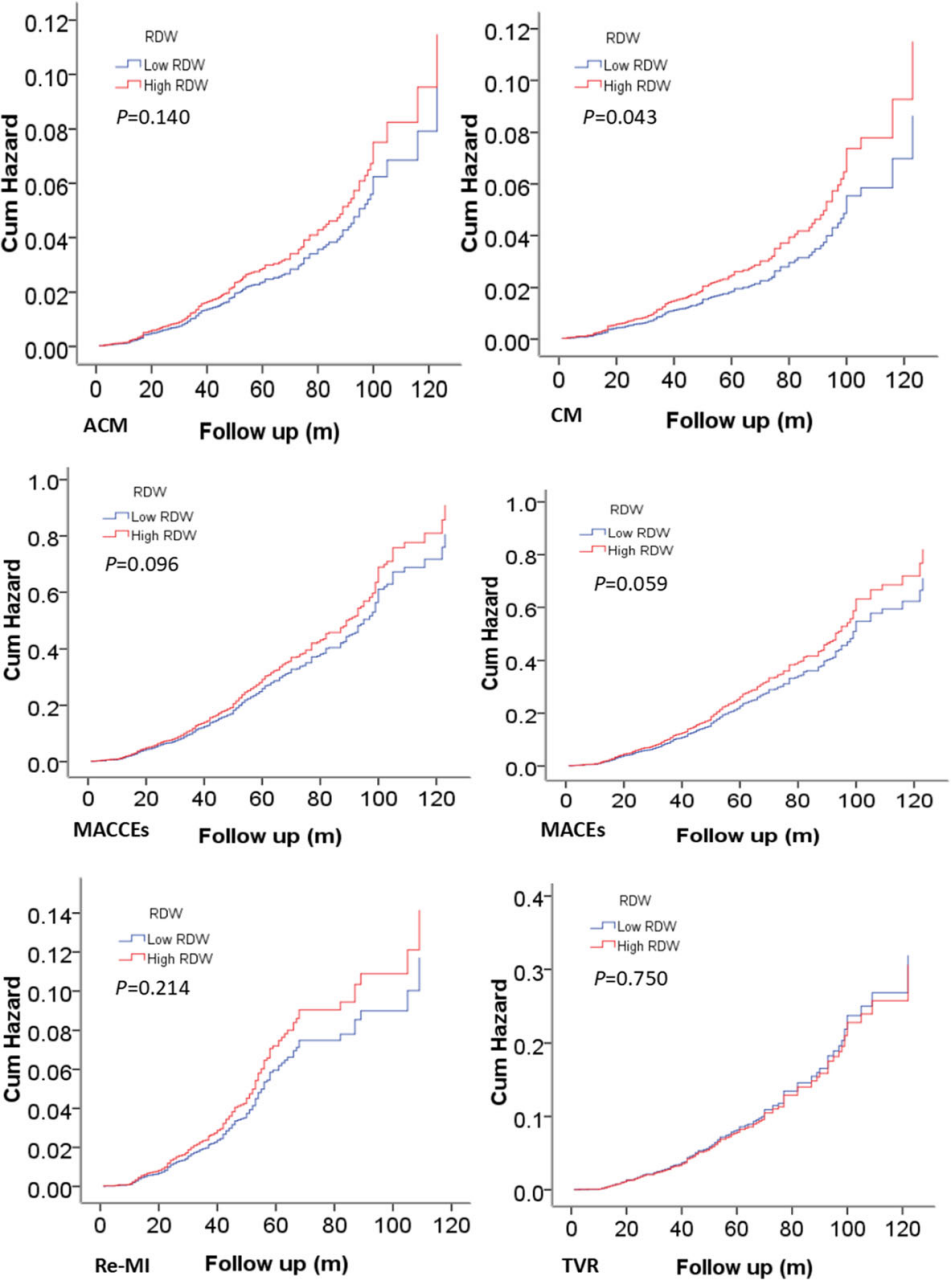
Cumulative Kaplan-Meier estimates of the time to the first adjudicated occurrence of primary endpoint and secondary endpoints

Kaplan–Meier survival analysis suggested that patients with high RDW exhibited increased accumulated risk of cardiac death (Fig. 2).

## Discussion

The present studies investigated the prognostic value of RDW in CAD patients with PCI and found that RDW was an independent poor prognosis factor at levels ≥13.1%. High levels increased long-term cardiac death 1.33 times. To reduce the risk of confounding, we adjusted for a comprehensive list of characteristics that influence the risk of cardiovascular events to examine the association between RDW and clinical outcomes. Uyarel et al. [22] retrospectively enrolled 2506 consecutive acute myocardial infarction patients undergoing primary PCI and found a significant association between elevated admission RDW levels and the adjusted risk of cardiovascular mortality (HR: 1.831, 95% CI: 1.034–3.24, *P* = 0.03) after a mean period of 1.8 ± 1.3 years. Fatemi et al. [23] enrolled 1689 patients from four centers who underwent PCI and found that RDW was an independent correlate of 1-year mortality (HR 1.65, CI 1.22–2.23, *P* = 0.001). Several studies also found a high baseline RDW value in patients with specific baseline characteristics, such as non-anemic at baseline, [24] diabetic [25] or elderly CAD [26] undergoing primary PCI was independently associated with an increased risk for long-term all-cause mortality, and it a prognosis risk factor for MACE in acute coronary syndrome patients [27, 28]. Our multifactorial Cox regression analysis showed that increased RDW was an independent risk factor for poor prognosis during the follow-up period, which was reported previously. We found that higher RDW was independently associated with adverse outcomes. When RDW was higher than 13.1, the incidence of cardiac death increased 1.33 times. Our study has some advantages over previous studies. First, the large sample size was a strength of our study, which improved the statistical power. Second, all patients were CAD after PCI and were followed up for up to 10 years. This follow-up duration was longer compared to previous studies.

Higher levels of RDW within the normal range may indicate accelerated red blood cell destruction or, more commonly, ineffective erythropoiesis. The identification of a putative mechanism is hampered by the lack of epidemiological studies demonstrating the factors that are associated with anisocytosis [20]. The exact mechanism associated with the prognosis of RDW and cardiovascular disease is not clear. However, related literature reports that increased RDW may be a comprehensive reflection of various mechanisms in the pathophysiological process. A number of studies have shown that inflammatory factors, such C-reactive protein (CRP), interleukin 6, fibrinogen level and white blood cell count alter erythrocyte homeostasis and may contribute to increased RDW levels by impairing iron metabolism, inhibiting the production or response to erythropoietin or by shortening red blood cell survival. Pro-inflammatory cytokines suppress erythropoietin gene expression, inhibit the proliferation of erythroid progenitor cells, down-regulate erythropoietin receptor expression, and reduce erythrocyte life-span [29–32].

In addition to inflammation, oxidative stress may be another important mechanism to explain the underlying relationship. Oxidative damage is prone to reduce cell survival and have a disadvantage on the homogeneity of erythrocytes. As the primary “oxidative sink”, erythrocytes have tremendous antioxidant capacity, and the variation in erythrocyte size is associated with oxidative stress, likely via increased red blood cell turnover [33]. Bujak et al. [34] clarified the prognostic role of RDW in CAD, and they insist that the prognostic value of RDW primarily results from the negative impact of inflammation oxidative stress and iron and vitamin D_3_ deficiency on bone marrow erythropoiesis. The concurrent red blood cell deformability diminution may result in impaired flow through the microcirculation. It is impossible to unambiguously ascertain which concomitant factors, including lipid, glycemic and iron metabolism disturbances, anemia, vitamin D_3_ deficiency, oxidative stress, inflammation, and the diminution of erythrocyte membrane deformability, are the primary causes of the poor prognoses observed in patients with CAD.

### Limitations

Our study has some limitations that should be considered. First, this study failed to monitor and measure inflammation changes, neurohumoral markers, including C-reactive protein, brain natriuretic peptide and other pro-inflammatory cytokines, angiotensin II and norepinephrine levels, and these indicators may better explain the higher RDW values, which are increased in patients with malignant mechanism, on the risk of cardiac events. Second, the present study was a single retrospective cohort design. Therefore, our results must be further verified in a multicenter, prospective study to confirm the association between RDW and adverse outcomes.

## Conclusions

In conclusion, our findings are notable because RDW is widely available to clinicians as part of the complete blood count, incurs no additional costs, and may be a novel marker of cardiovascular prognosis.

## Data Availability

Due to confidentiality policies, the data will not be shared.

## Abbreviations

ACM: All-cause mortality
CAD: Coronary artery disease
CAG: Coronary angiography
LVEF: Left ventricular ejection fraction
MACE: Major adverse cardiovascular events
RDW: Red blood cell distribution width

## References

[1] Cai G, Shi G, Xue S, Lu W (2017). The atherogenic index of plasma is a strong and independent predictor for coronary artery disease in the Chinese Han population. Medicine (Baltimore).

[2] Sniderman AD, Williams K, Contois JH, Monroe HM, McQueen MJ, de Graaf J, Furberg CD (2011). A meta-analysis of low-density lipoprotein cholesterol, non-high-density lipoprotein cholesterol, and apolipoprotein B as markers of cardiovascular risk. Circ Cardiovasc Qual Outcomes.

[3] Sierra-Johnson J, Fisher RM, Romero-Corral A, Somers VK, Lopez-Jimenez F, Ohrvik J, Walldius G, Hellenius ML, Hamsten A (2009). Concentration of apolipoprotein B is comparable with the apolipoprotein B/apolipoprotein A-I ratio and better than routine clinical lipid measurements in predicting coronary heart disease mortality:findings from a multi-ethnic US population. Eur Heart J.

[4] Aksu H, Ozer O, Unal H, Hobikoglu G, Norgaz T, Buturak A, Soylu O, Narin A (2009). Significance of mean platelet volume on prognosis of patients with and without aspirin resistance in settings of non-ST-segment elevated acute coronary syndromes. Blood Coagul Fibrinolysis.

[5] Li G, Hou X, Li Y, Zhang P, Zhao Q, Li J, Shi J (2017). Prognostic value of glycated hemoglobin among patients with ST-segment elevation myocardial infarction: a systematic review and meta-analysis. Clin Chem Lab Med.

[6] Troughton JA, Woodside JV, Young IS, Arveiler D, Amouyel P, Ferrières J, Ducimetière P, Patterson CC, Kee F, Yarnell JW, Evans A, PRIME Study Group (2007). Bilirubin and coronary heart disease risk in the prospective epidemiological study of myocardial infarction (PRIME). Eur J Cardiovasc Prev Rehabil.

[7] Brie D, Sahebkar A, Penson PE, Dinca M, Ursoniu S, Serban MC, Zanchetti A, Howard G, Ahmed A, Aronow WS, Muntner P, Lip GY, Wong ND, Rysz J (2016). Banach M; lipid, blood pressure meta-analysis collaboration (LBPMC) group. Effects of pentoxifylline on inflammatory markers and blood pressure: a systematic review and meta-analysis of randomized controlled trials. J Hypertens.

[8] Ge C, Ren F, Lu S, Ji F, Chen X, Wu X (2009). Clinical prognostic significance of plasma cystatin C levels among patients with acute coronary syndrome. Clin Cardiol.

[9] Bautista LE, Arenas IA, Peñuela A, Martínez LX (2002). Total plasma homocysteine level and risk of cardiovascular disease: a meta-analysis of prospective cohort studies. J Clin Epidemiol.

[10] Felker GM, Allen LA, Pocock SJ, Shaw LK, McMurray JJ, Pfeffer MA, Swedberg K, Wang D, Yusuf S, Michelson EL, Granger CB, CHARM Investigators (2007). Red cell distribution width as a novel prognostic marker in heart failure: data from the CHARM program and the Duke databank. J Am Coll Cardiol.

[11] Emans ME, Gaillard CA, Pfister R, Tanck MW, Boekholdt SM, Wareham NJ, Khaw KT (2013). Red cell distribution width is associated with physical inactivity and heart failure, independent ofestablished risk factors, inflammation or iron metabolism; the EPIC-Norfolk study. Int J Cardiol.

[12] Van Craenenbroeck EM, Conraads VM, Greenlaw N, Gaudesius G, Mori C, Ponikowski P, Anker SD (2013). The effect of intravenous ferric carboxymaltose on red cell distribution width: a subanalysis of the FAIR-HF study. Eur J Heart Fail.

[13] Pascual-Figal DA, Bonaque JC, Redondo B, Caro C, Manzano-Fernandez S, Sánchez-Mas J, Garrido IP, Valdes M (2009). Red blood cell distribution width predicts long-term outcome regardless of anaemia status inacute heart failure patients. Eur J Heart Fail.

[14] Adamsson Eryd S, Borné Y, Melander O, Persson M, Smith JG, Hedblad B, Engström G (2014). Red blood cell distribution width is associated with incidence of atrial fibrillation. J Intern Med.

[15] Lee KH, Park HW, Cho JG, Yoon NS, Kim SS, Kim MR, Kim MC, Cho KH, Kim HK, Kim CH, Kim KH, Jun SJ, Kim WJ, Lee KJ, Jeong HC, Cho JY, Park KH, Ds S, Yoon HJ, Kim KH, Hong YJ, Kim JH, Ahn Y, Jeong MH, Park JC (2015). Red cell distribution width as a novel predictor for clinical outcomes in patients with paroxysmalatrial fibrillation. Europace.

[16] Yanagisawa S, Inden Y, Kato H, Miyoshi A, Mizutani Y, Ito T, Kamikubo Y, Kanzaki Y, Hirai M, Murohara T (2016). Elevated red blood cell distribution width predicts recurrence after catheter ablation for atrial fibrillation in patients with heart failure - comparison with non-heart failure patients. Circ J.

[17] Rhodes CJ, Wharton J, Howard LS, Gibbs JS, Wilkins MR (2011). Red cell distribution width outperforms other potential circulating biomarkers in predicting survival in idiopathic pulmonary arterial hypertension. Heart.

[18] Zorlu A, Bektasoglu G, Guven FM, Dogan OT, Gucuk E, Ege MR, Altay H, Cinar Z, Tandogan I, Yilmaz MB (2012). Usefulness of admission red cell distribution width as a predictor of early mortality in patients with acute pulmonary embolism. Am J Cardiol.

[19] Ye Z, Smith C, Kullo IJ (2011). Usefulness of red cell distribution width to predict mortality in patients with peripheral artery disease. Am J Cardiol.

[20] Tonelli M, Sacks F, Arnold M, Moye L, Davis B, Pfeffer M (2008). For the cholesterol and recurrent events (CARE) trial investigators. Relation between red blood cell distribution width and cardiovascular event rate in people with coronary disease. Circulation.

[21] Osadnik T, Strzelczyk J, Hawranek M, Lekston A, Wasilewski J, Kurek A, Gutowski AR, Wilczek K, Dyrbuś K, Gierlotka M, Wiczkowski A, Gąsior M, Szafranek A, Poloński L (2013). Red cell distribution width is associated with long-term prognosis in patients with stable coronary artery disease. BMC Cardiovasc Disord.

[22] Uyarel Huseyin, Ergelen Mehmet, Cicek Gokhan, Kaya Mehmet Gungor, Ayhan Erkan, Turkkan Ceyhan, Yildirim Ersin, Kirbas Veli, Onturk Ebru Tekbas, Erer Hatice Betul, Yesilcimen Kemal, Gibson C. Michael (2011). Red cell distribution width as a novel prognostic marker in patients undergoing primary angioplasty for acute myocardial infarction. Coronary Artery Disease.

[23] Fatemi Omid, Paranilam Jaya, Rainow Alex, Kennedy Kevin, Choi Jason, Cutlip Donald, Pencina Michael, Berger Peter B., Cohen David J., Kleiman Neal S. (2012). Red cell distribution width is a predictor of mortality in patients undergoing percutaneous coronary intervention. Journal of Thrombosis and Thrombolysis.

[24] Poludasu Shyam, Marmur Jonathan D., Weedon Jeremy, Khan Waqas, Cavusoglu Erdal (2009). Red cell distribution width (RDW) as a predictor of long-term mortality in patients undergoing percutaneous coronary intervention. Thrombosis and Haemostasis.

[25] Tsuboi Shuta, Miyauchi Katsumi, Kasai Takatoshi, Ogita Manabu, Dohi Tomotaka, Miyazaki Tadashi, Yokoyama Takayuki, Kojima Takahiko, Yokoyama Ken, Kurata Takeshi, Daida Hiroyuki (2013). Impact of Red Blood Cell Distribution Width on Long-Term Mortality in Diabetic Patients After Percutaneous Coronary Intervention. Circulation Journal.

[26] Liu XM, Ma CS, Liu XH, Du X, Kang JP, Zhang Y, Wu JH. Relationship between red blood cell distribution width and intermediate-term mortality in elderly patients after percutaneous coronary intervention. J Geriatr Cardiol. 2015;12(1):17–22. 10.11909/j.issn.1671-5411.2015.01.013 PMC4308454 25678900

[27] Zhao Na, Mi Lan, Liu Xiaojun, Pan Shuo, Xu Jiaojiao, Xia Dongyu, Liu Zhongwei, Zhang Yong, Xiang Yu, Yuan Zuyi, Guan Gongchang, Wang Junkui (2015). Combined Value of Red Blood Cell Distribution Width and Global Registry of Acute Coronary Events Risk Score for Predicting Cardiovascular Events in Patients with Acute Coronary Syndrome Undergoing Percutaneous Coronary Intervention. PLOS ONE.

[28] Isik Turgay, Kurt Mustafa, Tanboga Ibrahim Halil, Ayhan Erkan, Gunaydin Zeki Yuksel, Kaya Ahmet, Uyarel Huseyin (2016). The impact of admission red cell distribution width on long-term cardiovascular events after primary percutaneous intervention: A four-year prospective study. Cardiology Journal.

[29] Rechavi G, Rivella S (2008). Regulation of iron absorption in hemoglobinopathies. Curr Mol Med.

[30] Weiss G, Goodnough LT (2005). Anemia of chronic disease. N Engl J Med.

[31] Veeranna V, Zalawadiya SK, Panaich S, Patel KV, Afonso L (2013). Comparative analysis of red cell distribution width and high sensitivity C-reactive protein forcoronary heart disease mortality prediction in multi-ethnic population: findings from the 1999-2004 NHANES. Int J Cardiol.

[32] Schieffer B, Schieffer E, Hilfiker-Kleiner D, Hilfiker A, Kovanen PT, Kaartinen M, Nussberger J, Harringer W, Drexler H (2000). Expression of angiotensin II and interleukin 6 in human coronary atherosclerotic plaques:potential implications for inflammation and plaque instability. Circulation.

[33] Friedman JS, Lopez MF, Fleming MD, Rivera A, Martin FM, Welsh ML, Boyd A, Doctrow SR, Burakoff SJ (2004). SOD2-deficiency anemia: protein oxidation and altered protein expression reveal targets ofdamage, stress response, and antioxidant responsiveness. Blood.

[34] Bujak K, Wasilewski J, Osadnik T, Jonczyk S, Kołodziejska A, Gierlotka M, Gąsior M (2015). The prognostic role of red blood cell distribution width in coronary artery disease: a review of the pathophysiology. Dis Markers.

